# Openness as infrastructure

**DOI:** 10.1186/1758-2946-3-36

**Published:** 2011-10-14

**Authors:** John Wilbanks

**Affiliations:** 1 VP of Science, Creative Commons, San Francisco, California, USA

## Abstract

The advent of open access to peer reviewed scholarly literature in the biomedical sciences creates the opening to examine scholarship in general, and chemistry in particular, to see where and how novel forms of network technology can accelerate the scientific method. This paper examines broad trends in information access and openness with an eye towards their applications in chemistry.

## Commentary

Science, like culture, is grounded in stories. Science has long sought to make sense of the information we receive from the world around us, resolved to tell stories that are supported by data, that explain why the sun comes up in the east, and goes down in the west, and does so every day, without recourse to mystical beings. And the way we communicate science belongs to this long storytelling tradition: we write papers, and publish them, so that others might know the stories of what happened in a given laboratory at a given time, that someone found the crystal structure of DNA, or that light behaves like a wave and a particle at the same time.

These stories are validated by their presence in journals, collections of stories, bound up and published monthly, many physically printed and mailed out even in the digital age. If the story is in a famous journal, it's trustable. If the story is in John's Journal Of Chicken-Fried Science-or, less facetiously, a journal that is bought and paid for by a pharmaceutical company [1]-it is not. This trust comes from the brand of the journal, built over the years through the recruitment of trusted scientists to serve as peer reviewers. And this entire method encases the idea that individual scientists, the principal investigators, are romantic entities at the core of the laboratory, shouting Eureka and running naked through the halls after proving a new theorem.

The truth is of course a lot more complex. Principal investigators depend on postdoctoral and graduate students. The paper is merely an advertisement for years of research(Endnote 1), a snapshot of a far more complex knowledge generation process, but for hundreds of years, it's been the best knowledge compression technology available to us. The papers have become finely tuned objects where some of the text is used to show the author understands the existing paradigm of the field, some of the text is used to describe the methods and results, and some is used to describe the implications. Each of these sections needed to be terse, as paper was expensive to print and ship.

This hid the fact that science was, in fact, actually much more like a wiki. Every topic in science is open for back and forth, and new discoveries spark rounds of editing and re-editing, and the print equivalent of flame wars in biting letters to the editor. But it was a wiki that was camouflaged as physical media. And in an era of increasingly computerized science, with automated and massively parallel lab equipment pumping data into massively parallel processing power, we're starting to see an absolutely overwhelming increase in the number of digital papers. Leaving behind the irony of digital paper, there is a strong parallel in science today to when cities crested ahead of their sewer systems and highways-industrial knowledge production capacity, pre-colombian recycling capacity. Science is drowning in its own outputs, and a lot of those outputs are turning out to be either non-reproducible [2] or downright false [3].

What we need is a full-scale revolution in the way that we publish knowledge, and there are many claimaints to carry the standard of that revolution. Some are from the "radical incrementalism" school(Endnote 2)-into which I would put Open Access, a movement that puts literature online, free of charge, and free of copyright restrictions other than providing credit to the author (there are several core definitions of Open Access, but I am quoting here from the Budapest Open Access Initiative [4]), as well as the movement to separate the subjective judgement of impact from a more objective judgement of scientific validity in the peer review process [5]. Others go farther, arguing for the abandonment of the article as the core unit of knowledge transfer, for nano-publication of individual assertions [6], for the publication of figures or data rather than articles [7], for the rise of wiki science and the end of peer review entirely [8].

It's an explosion in our capacity to capture data that is a large player in the explosion of papers, and in the various claimants to the revolution in publishing knowledge. We now have massively parallel ways to measure reactions, run experiments, capture information about the state of the world. But the publication revolution (that is, beyond radical incrementalism) will not occur without some new help. The promised Fourth Paradigm of Science [9] will require that we build new systems into the existing *data *infrastructure that we have for science.

Infrastructure used to be something physical-highways, in the common world, or big buildings and expensive machines in the science world (such as the Large Hadron Collider, or the Hubble Telescope). The rise of the network has brought a new layer of physical infrastructure, from the fiber across which bits flow to the server farms and compute clusters and clouds where processing now takes place, all connected by yet another crucial element-the standard protocols by which data and documents and music files and more are broken up into packets, routed, transported, and reassembled. And one of the most important sets of protocols is the set we know generally speaking as the Web. It's the stuff that lets us share documents, and it's changed the world.

But in the case of complex adaptive systems-like the body, the climate, or our national energy usage-the data are usually not part of a document. They exist in massive databases which are loosely coupled, and are accessed by humans not through search engines but through large-scale computational models. There are so many layers of abstraction between user and data that it's often hard to know where the actual data at the base of a a set of scientific claims reside.

This is at odds with the fundamental nature of the Web. The Web is a web of documents. Those documents are all formatted the same way, using a standard markup language, and the same protocol to send copies of those documents around. Because the language allows for "links" between documents, we can navigate the Web of documents by linking and clicking. Because the right to link is granted to creators of web pages, we get lots of links. And because we get lots of links (and there aren't fundamental restrictions on copying the web pages) we get innovative companies like Google that index the links and rank web pages, higher or lower, based on the number of links referring to those pages [10]. Google doesn't know, in any semantic sense, what the pages are about, or what they mean. It simply has the power to do clustering and ranking at a scale never before achieved, and that turns out to be good enough.

But in the data world, very little of this applies. The data exist in a world almost without links. There is no accepted standard language, though some are emerging [11], to mark up data. And if you had that, then all you get is another problem-the problem of semantics and meaning. So far at least, the statistics aren't good enough to help us really structure data the way they structure documents.

There is one emerging world of data, often location-based data, where we can make a lot of progress. It's the world of apps that help you know when the bus will be at a given stop in Boston, and thus avoid the cold [12]. It's one that doesn't worry much about data integration, or data interoperability, or data infrastructure, because it's simple data-where is the bus and how fast is it going?-and because it's mapped against a knowledge system we have had for hundreds of years, that we understand, and which is...well, a map.

But the world of modern science isn't so simple. Doing deeply complex modeling of climate events, of energy usage, of cancer progression-these are not so easy to turn into iPhone apps. The way we treat them shouldn't be with the output of a document. It's the wrong metaphor. We don't need a "map" of cancer-at least not in the classical sense of a 2-dimensional representation. We need a model that tells us, given certain inputs, what our decision matrix looks like. And the infrastructure for documents doesn't get us there.

So, I have made the argument for more infrastructure. That imposes the requirement that I say what I mean by infrastructure. I believe there to be at least three essential elements missing.

First is the infrastructure to collaborate scientifically. Laboratories are natural breeding grounds for collaboration and conversation-reagents are shared, coffee and tea are drunk, journal club is hosted. Virtual collaboration lacks these elements that form the circadian rhythms of a group, and this absence of shared rhythm dogs collaborative projects far beyond the sciences (Endnote 3). We have seen some infrastructure for distributed collaboration in software, like github, but as yet this has not emerged in the sciences (and indeed may need to evolve discipline by discipline as needs and local context dictate).

Another missing link is that of classification. Before the web, classification was a library or taxonomical function, imposed from above by hierarchical authority, famously subject to bias, prejudice, and sheer incompetence (Endnote 4). But with the advent of the web, we see the rise of "categories, links, and tags" as emergent systems of classification, ones that are plenty good enough to help us fine web pages about ourselves, ratings of local restaurants, or lengthy rants against ontology. We no longer need a file system, we just need the right search string (and of course, services that provide us the search capacity).

But science actually fits many of the elements where expert classification and formal ontology actually make some sense-formal categories, expert users, authoritative sources of judgement, etc. And in particular, the problem that automated machine-generated data imposes of an explosion of unstructured content means that the emergent classification on which the Web runs doesn't emerge, *because there aren't any people tagging it and linking it*. We have to have at least some formal classifications to impose to help us deal with big data, but science doesn't like to fund that sort of work nearly as much as it does the creation of new (you guessed it) papers.

The last one is thankfully the easiest of the three. It is the infrastructure for *data openness*. It's composed of open data (Endnote 5) licenses (Endnote 6)(covering not only copyright and database rights, but issues of privacy, identity, and more (Endnote 7)), legal user interfaces to make sure users understand the terms, and technological implementations for licenses, so that machines can negotiate and discover the terms under which a given piece of data is (or isn't) available. This infrastructure for openness draws on successes in free software and free culture, where open licenses have been part of the creation of entire ecosystems of co-creation that would otherwise have been impossible [13].

Open data also helps us address the first two elements of missing infrastructure. It's highly unlikely that any one scientific funder, or any one company, will develop the right system for collaboration across sciences, or even across a single discipline in the sciences like chemistry. Open data means that the disciplines can each evolve towards their own systems of collaboration, that the marketplace of ideas can take place without high transaction costs to try, and often fail, at new methods to work together. Open data also helps address the classification problem, again by lowering the cost at which one group attempts to organize their information, and by creating a culture in which classification schemes are themselves shared, remixed, hacked, and subjected to incremental improvement-but also ready to be torn down and rebuilt when the data indicate.

There are two striking examples of open data that we can look to as inspiration for chemistry. One is in astronomy, where there is a longstanding tradition (caused in part by scarce, and thus shared, physical resources like radio telescopes) of sharing open data, as well as an evolved, open source infrastructure for virtual collaboration (Endnote 8). Openness has become the norm, and has allowed for classification and collaboration to emerge over time, so that now the serious work of astronomical science takes place in the open.

A second is more emergent, and more scattered, in biology. Biology has for years been like chemistry-laboratory focused, principal investigator driven-and subject to enormous competitive pressures with the boom of the biotechnology industry. But the larger the data become, and the more complex the human body is discovered to be, the more open data becomes the only tractable methodological approach that accelerates science. Chemistry itself is seeing a flowering of expertise in novel methods of publication and knowledge construction, though only time will tell which approach will become part of the infrastructure of the discipline (Endnote 9).

Thus, the pharmaceutical industry itself has systematically invested in the public domain of data, from the Single Nucleotide Polymorphism Consortium (Endnote 10) to the Structural Genomics Consortium (Endnote 11). As the pharmaceutical industry is well known to embrace patent rights in many areas, its decade-long investment in, and support of, open data is a telling example of the market finding its own way towards openness as infrastructure that simply accelerates science. The recent advent of Sage Bionetworks, another non profit data sharing project, promises to bring the same kind of benefits to disease biology, moving from "fundamental" data like sequences and structures to experimental and clinical information.

Taken together, these three skeins of collaboration, classification, and openness draw us inevitably towards the long-claimed, but rarely-achieved, goal of the scientific method: to make claims that are reproducible under similar circumstances by someone other than the claimant, to be reproducible.

The road to implementing the three new levels of data infrastructure face barriers. Science is complex, and even if we implement on all three levels, that won't magically create new insights. The Alzheimer's Disease Neuroimaging Initiative ran for nearly a decade as an open data, open collaboration project, with standardized ways to classify the images, before its research breakthroughs made it into the peer-reviewed (wait for it) papers [14]. There is a lag time between when we invest in infrastructure and when we see the results, and we will have to be patient.

But open data will in the end win out, just as open systems have won out for networking, for document sharing, for software, and are beginning to win for culture and education. It is, in the end, the better way to do science, one in which there is less duplication of effort, less fraud, more reproducibility, more return on investment, and faster times to market of knowledge. It is, moreover, one that returns scientific data to its most natural state, one that is a pure public good, that gains more value as more people possess it.

## Endnotes

### Endnote 1

Apocryphal, but told to the author by Victoria Stodden.

### Endnote 2

I owe this phrase to a conversation with Christine Borgman of the University of California of Los Angeles.

### Endnote 3

See The World Opera project for a fascinating example at http://theworldopera.org/- debates that never occur in a normal opera, such as "should we have a real conductor at one location, an avatar, or just a metronome?" must be resolved before a collaborative performance in real time can be achieved.

### Endnote 4

Clay Shirky has written a lovely deconstruction of classification called "Ontology is Overrated"-available at http://www.shirky.com/writings/ontology_overrated.html. This paragraph draws on his arguments at multiple points, but I encourage readers to read the whole article, including his high praise of the periodic table of the elements as a high-water mark in classification.

### Endnote 5

See the Open Knowledge Definition at http://www.opendefinition.org/okd/- although I dispute the idea that data necessarily equals knowledge, I still like the definition's spirit.

### Endnote 6

See Creative Commons' CC0 legal tool at http://creativecommons.org/publicdomain/zero/1.0/ for an example of an implementation of the OKD for data.

### Endnote 7

This is a space where the naive "porting" of open infrastructure for software and culture fails. Privacy constraints, especially around human subjects data, are totally orthogonal to the right to make and distribute copies. This is a key area for future work and research.

### Endnote 8

See the International Virtual Observatory Alliance, at http://www.ivoa.net/, for a remarkable example of international virtual science based on public domain data.

### Endnote 9

For example, http://www.openphacts.org/, http://semanticweb.com/semantic-chemistry_b10684, http://en.wikipedia.org/wiki/Blue_Obelisk, http://en.wikipedia.org/wiki/Open_Notebook_Science, http://chem2bio2rdf.org

### Endnote 10

The SNP Consortium (TSC) was established in 1999 as a collaboration of several companies and institutions to produce a public resource of single nucleotide polymorphisms (SNPs) in the human genome. The initial goal was to discover 300 000 SNPs in two years, but the final results exceeded this, as 1.4 million SNPs had been released into the public domain at the end of 2001. In the end, 1.8 million SNPs were released. More than $50,000,000 was contributed to fund this project, the majority by for-profit companies from [15] and from "The SNP Fact Sheet" at http://www.ornl.gov/sci/techresources/Human_Genome/faq/snps.shtml.

### Endnote 11

The SGC is a public-private partnership whose mandate is to promote the development of new medicines by carrying out basic science of relevance to drug discovery and placing all information, reagents and know-how into the public domain without restriction. The core mandate of the SGC is to determine 3D structures on a large scale and cost-effectively-targeting human proteins of biomedical importance and proteins from human parasites that represent potential drug targets. In these two areas, the SGC is now responsible for >25% and >50% of all structures deposited into the Protein Data Bank each year. It is funded by public and private institutions, including three of the world's largest pharmaceutical companies. From the SGC FAQ at http://www.thesgc.org/about/faqs.php#faq_3
